# Primary osseous kaposiform hemangioendothelioma confined to long bones: a retrospective imaging analysis of 12 pediatric cases

**DOI:** 10.1186/s40644-026-01002-2

**Published:** 2026-02-12

**Authors:** Zhenliang Hao, Dalin Gao, Wei Zhang, Shan Huang, Dalong Gu, Fan Yang, Wei Zhang, Yi Ding, Dong Yan

**Affiliations:** 1 Department of Radiology, Beijing Jishuitan Hospital, Capital Medical University, No. 68 Huinanbei Road, Huilongguan, Changping District, Beijing, 102208 China; 2 Department of Pathology, Beijing Jishuitan Hospital, Capital Medical University, No. 68 Huinanbei Road, Huilongguan, Changping District, Beijing, 102208 China

**Keywords:** Kaposiform hemangioendothelioma, Primary intraosseous tumor, Pediatric bone disease, Imaging patterns, Permeative bone destruction, Cortical excavation, Differential diagnosis

## Abstract

**Background:**

Kaposiform hemangioendothelioma (KHE) is a rare, locally aggressive vascular tumor predominantly affecting infants and children. While musculoskeletal involvement is common, cases originating in and confined exclusively to bone are exceptionally rare, and their specific imaging characteristics are not well-defined. This study aims to characterize the clinical and detailed radiological features of primary, intraosseous KHE in a pediatric cohort.

**Methods:**

We conducted a retrospective review of pediatric patients with biopsy-proven KHE admitted to our institution between January 2016 and December 2024. Inclusion criteria stipulated that lesions must be confined entirely within the bone on initial diagnostic imaging. The clinical data, computed tomography (CT), and magnetic resonance imaging (MRI) scans of 12 eligible patients were systematically analyzed by two senior musculoskeletal radiologists. Ki-67 proliferation index was analyzed to correlate with imaging patterns.

**Results:**

The cohort included 12 patients (11 males, 1 female) with a median age of 5 years (range, 2–12 years). Common presenting symptoms were chronic pain, limping, and muscle atrophy. Notably, no patients presented with Kasabach-Merritt phenomenon (KMP) or cutaneous lesions. Two distinct and recurring radiological patterns were identified. The first, a **permeative/moth-eaten pattern (*n* = 7/12)**, was characterized by dotted, infiltrative osteolytic lesions involving both the epiphysis and metaphysis, often with associated tubular formations. The second, a **cortical excavation pattern (*n* = 5/12)**, presented as well-defined, saucer-like erosions of the bone cortex, primarily in the metaphysis. No cases exhibited significant periosteal reaction or a discrete soft-tissue mass. Histopathology confirmed infiltrative nodules of spindled endothelial cells forming slit-like vascular channels. The Ki-67 proliferation index was significantly higher in Pattern 1 compared to Pattern 2 (18.7% vs. 9.2%, *P* < 0.001, excluding one mixed-pattern case).

**Conclusion:**

Primary KHE confined to bone presents with two distinct imaging patterns: permeative/moth-eaten infiltration and cortical excavation. These patterns correlate with biological aggressiveness as indicated by Ki-67 expression. Recognition of these specific features, particularly in the absence of KMP or skin changes, is crucial for radiologists and clinicians. These findings likely represent the earliest detectable stage of osseous KHE and should prompt consideration of this rare entity in the differential diagnosis of pediatric lytic bone lesions, facilitating timely diagnosis and intervention.

## Introduction

Kaposiform hemangioendothelioma (KHE) is a rare vascular tumor of intermediate malignancy, characterized by its locally aggressive and infiltrative nature [1, 2]. It predominantly occurs during infancy and early childhood, with most lesions located in the soft tissues of the extremities, trunk, retroperitoneum, and mediastinum [3–6]. Histopathologically, KHE is composed of nodules of spindled endothelial cells forming slit-like vascular channels. It is immunopositive for vascular (CD31, CD34) and lymphatic (D2-40/podoplanin) endothelial markers and negative for glucose transporter-1 (GLUT-1) and human herpesvirus-8 (HHV-8), distinguishing it from other vascular anomalies [7]. Notably, in the 2020 WHO classification of soft tissue and bone tumors, KHE is categorized primarily as a soft tissue vascular tumor and is not explicitly included in the classification of primary bone vascular tumors. This absence underscores the extreme rarity and lack of recognition of primary osseous KHE, highlighting the novelty and necessity of our study to characterize this distinct clinical entity.

A significant and life-threatening complication associated with KHE is the Kasabach-Merritt phenomenon (KMP), a severe consumptive coagulopathy involving profound thrombocytopenia, hypofibrinogenemia, and microangiopathic hemolytic anemia [8]. Cutaneous manifestations, such as purpuric or violaceous skin changes and indurated masses, are also common [8]. The underlying pathophysiology involves dysregulation of angiogenesis and lymphangiogenesis, primarily through the PI3K/Akt/mTOR signaling pathway, which has made mTOR inhibitors like sirolimus a cornerstone of effective therapy [9].

Although musculoskeletal system involvement in KHE has been reported in up to 62.8% of patients, these cases typically involve extensive soft-tissue masses with secondary bone invasion [10, 11]. In contrast, KHE originating primarily within bone and remaining strictly confined to the osseous compartment is an exceptional occurrence [12–14]. This specific presentation represents a diagnostic challenge, as its imaging features are not extensively documented and can mimic a range of other pediatric bone pathologies, from inflammatory conditions to malignant tumors. The absence of typical systemic signs like KMP or skin lesions in these early, localized cases further complicates the diagnostic process.

Recent studies have begun to classify KHE with bone involvement into two categories: bone-only lesions and lesions affecting both bone and surrounding soft tissue [15]. This distinction suggests that purely intraosseous KHE may represent the earliest stage of the disease spectrum. A thorough characterization of its initial imaging presentation is therefore critical for early detection, which can enable timely intervention and potentially prevent progression to more extensive disease associated with higher morbidity. The purpose of this study was to systematically analyze and characterize the detailed clinical and imaging findings in a cohort of pediatric patients with primary KHE strictly confined to bone, aiming to identify specific radiological patterns that can aid in its early diagnosis.

## Methods

### Study design and patient cohort

This retrospective case series was conducted in accordance with the principles of the Declaration of Helsinki. The study protocol was approved by the Institutional Review Board of Beijing Jishuitan Hospital. Written informed consent was waived due to the retrospective nature of the study. This report was prepared following the methodological principles recommended by the STROBE statement for observational studies and the PROCESS guidelines for surgical case series [16, 17].

We retrospectively reviewed the medical records and imaging database of our institution, a national orthopedic center, for pediatric patients (age < 18 years) diagnosed with KHE between January 2016 and December 2024. The inclusion criteria for this study were: (1) a definitive histopathological diagnosis of KHE obtained via biopsy or surgical resection; and (2) initial diagnostic imaging (CT and/or MRI) demonstrating lesions strictly confined to the osseous compartment without any associated extraosseous soft-tissue mass. Patients with primary soft-tissue KHE with secondary bone invasion or those with insufficient imaging data were excluded. A total of 12 consecutive patients met these criteria and were included in the analysis.

### Image acquisition

All patients underwent CT and/or MRI of the affected region. CT scans were performed using a Philips IQon Spectral CT scanner. The protocol included helical scanning with a slice thickness of 1 mm, a slice interval of 0.8 mm, and a pitch of 1.0. Scan parameters were 120 kV and 200–350 mA. Axial, coronal, and sagittal reconstructions were generated using standard bone and soft-tissue algorithms. When administered, the contrast agent was Iopromide (350 mgI/mL, Jiangsu Hengrui, China).

MRI scans were conducted on a Siemens Magnetom Vida 3.0T system. The imaging protocol was tailored to the specific anatomical location but typically included: axial and coronal T1-weighted spin-echo (SE) sequences (TR/TE, 415–750/16–40 ms); axial and coronal T2-weighted fast spin-echo (FSE) sequences (TR/TE, 2000–2500/100 ms); and sagittal or axial proton density-weighted fat-suppressed (PD-FS) sequences (TR/TE, 2300–3700/40–60 ms). Slice thickness was 3.0 mm with a 3.0 mm gap. For contrast-enhanced scans, gadopentetate dimeglumine (Gd-DTPA) was administered intravenously at a dose of 0.1 mmol/kg.

### Image analysis

All available CT and MR images were retrospectively and independently assessed by two senior musculoskeletal radiologists with over 10 and 15 years of experience, respectively. The radiologists were blinded to the final histopathological report at the time of initial review. Discrepancies in interpretation were resolved by a consensus review. The following imaging features were systematically evaluated:

- Lesion Location: Epiphysis, metaphysis, or diaphysis of the affected bone.
- Pattern of Bone Destruction: Classified according to the Lodwick classification as geographic, moth-eaten, or permeative.
- Margin Characteristics: Well-defined, ill-defined, sclerotic, or non-sclerotic.
- Internal Characteristics: Presence of cysts, calcifications, hemorrhage, or tubular/channel-like structures.
- Periosteal Reaction: Absent, or present (classified as solid, lamellated/onion-skin, or sunburst).
- Extraosseous Component: Presence or absence of a soft-tissue mass or adjacent fat stranding.
- MRI Signal Intensity: Signal characteristics on T1-weighted, T2-weighted, and fat-suppressed sequences.
- Enhancement Pattern: Pattern and degree of enhancement after contrast administration.

### Histopathological analysis

Biopsy or surgical specimens from all 12 patients were fixed in 10% neutral buffered formalin, decalcified where necessary, and embedded in paraffin. Sections were stained with hematoxylin and eosin (H&E). Immunohistochemical (IHC) staining was performed using standard protocols for vascular and lymphatic markers, including CD31, CD34, D2-40 (podoplanin), and smooth muscle actin (SMA), to confirm the diagnosis. The Ki-67 proliferation index was quantified as the percentage of positively stained nuclei in 1000 tumor cells in the most active areas (hot spots).

## Results

### Demographic and clinical findings

**Fig. 1:**
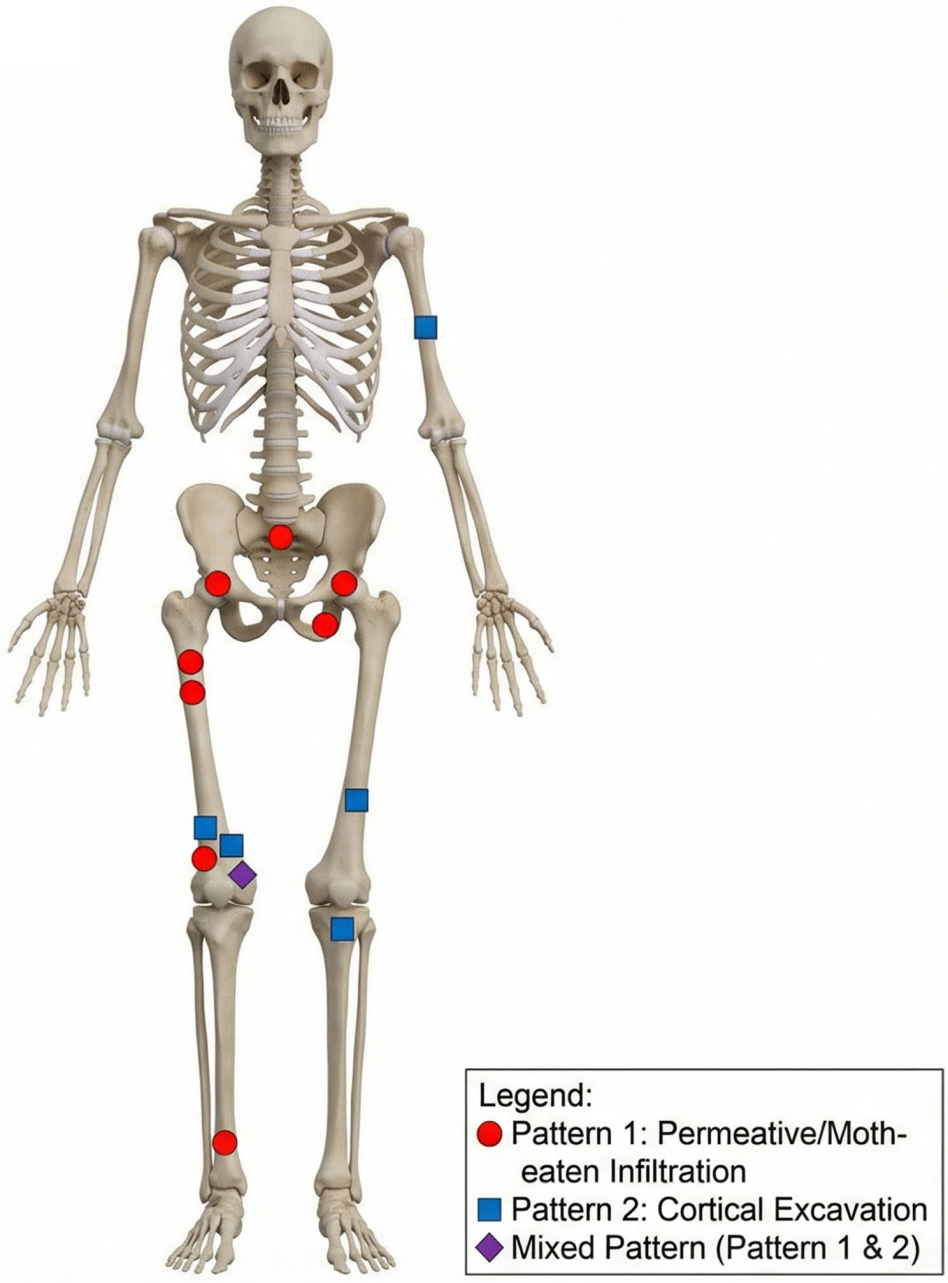
Anatomic distribution of primary osseous KHE lesions. Schematic representation of the human skeleton illustrating the specific locations of the 12 cases included in this study. The markers are coded by radiological pattern: red circles indicate the Permeative/Moth-eaten pattern (Pattern 1), blue squares indicate the Cortical Excavation pattern (Pattern 2), and the purple diamond indicates the Mixed pattern. The lesions were predominantly located in the lower extremities (femur and tibia)

The study cohort consisted of 12 children, with a notable male predominance (11 males, 1 female; ratio 11:1). The age at presentation ranged from 2 to 12 years, with a median age of 5 years. Patients were referred to our orthopedic center for a variety of nonspecific musculoskeletal symptoms, including chronic pain (*n* = 8), limping or abnormal gait (*n* = 6), decreased range of motion in the affected joint (*n* = 4), and localized muscle atrophy (*n* = 3). No patient exhibited purpuric or other cutaneous lesions, palpable masses, discharging sinuses, or significant regional lymphadenopathy. Laboratory findings, where available, showed no evidence of thrombocytopenia or coagulopathy, and none of the patients met the diagnostic criteria for KMP. All lesions were confined to the long bones of the extremities or the pelvic girdle. Detailed demographic and clinical data are summarized in Table 1. The anatomical distribution of the lesions is visually represented in Fig. 1.

**Table 1:** Summary of demographic, clinical, and radiological findings in 12 patients with primary osseous KHE

Case No.	Age (years)	Sex	Location	Clinical Symptoms	Radiological Pattern	Key Imaging Features	Ki-67 Index (%)
1	5	M	Bilateral acetabulum, pelvis, sacrum	Limping	Pattern 1: Permeative	Moth-eaten infiltration; tubular channels across planes.	20
2	4	M	Distal right femur (metaphysis)	Limping	Pattern 2: Excavation	Posterior cortical “apple bite” defect (1.6 × 0.7 cm) with irregular base.	8
3	8	M	Proximal right femur (epiphysis, metaphysis)	Abnormal gait, muscle atrophy	Pattern 1: Permeative	Dotted erosion in epiphysis; long tubular channel (5.9 cm) in metaphysis with mild sclerosis.	18
4	3	M	Distal right tibia	Abnormal gait	Pattern 1: Permeative	Permeative lesions forming a tubular channel (1.9 cm) with sclerosis; minimal fat stranding.	22
5	2	M	Distal right femur (epiphysis, metaphysis)	Pain	Mixed (Pattern 1 & 2)	Dotted lesions in epiphysis; cortical excavation (1.0 × 0.8 cm) in metaphysis; transphyseal channel.	15
6	4	M	Proximal right femur (epiphysis, metaphysis)	Pain	Pattern 1: Permeative	Dotted erosion in epiphysis/metaphysis; transphyseal channel (1.4 cm) with sclerosis.	17
7	7	M	Proximal left tibia (epiphysis)	Limping, pain, muscle atrophy	Pattern 2: Excavation	Cortical bite (1.6 × 0.9 cm) in epiphysis with irregular sclerosis; minimal cortical breach.	10
8	12	M	Proximal left humerus	Shoulder pain, worse at night	Pattern 2: Excavation	Cortical excavation (2.5 × 0.9 cm) with adjacent soft tissue edema.	7
9	10	M	Distal left femur (metaphysis)	Knee pain	Pattern 2: Excavation	Linear cortical excavation (2.5 × 0.3 cm).	9
10	8	M	Distal right femur (epiphysis, metaphysis)	Exercise-related pain, limping	Pattern 1: Permeative	Moth-eaten lesions forming multiplanar tubular structures.	19
11	7	M	Distal right femur (metaphysis)	Pain, limping	Pattern 2: Excavation	Multiple small cortical excavations (4–5 mm each).	12
12	6	F	Right acetabulum, ischium	Muscle atrophy	Pattern 1: Permeative	Moth-eaten infiltration; tubular channels across planes.	16

### Imaging findings

Based on the comprehensive radiological review, the 12 cases of primary osseous KHE could be categorized into two distinct and recurring imaging patterns (Table 2).

**Table 2:** Key differential diagnoses for primary osseous KHE based on imaging patterns

Condition	Similarities to KHE	Key Differentiating Features
**Differential for Pattern 1 (Permeative/Moth-eaten)**		
Ewing Sarcoma	Permeative osteolysis; pediatric age group.	Almost always has a large soft-tissue mass; prominent aggressive periosteal reaction (lamellated, sunburst). KHE lacks these.
Langerhans Cell Histiocytosis (LCH)	Lytic bone lesions in children.	Lesions are often “punched-out” with beveled edges; solid periosteal reaction is common. KHE is more infiltrative and crosses the physis.
Acute/Chronic Osteomyelitis	Lytic bone destruction.	**Acute**: Rapid onset, round lytic lesions, soft tissue swelling, signs of inflammation (fever, redness, heat, pain). **Chronic**: Prominent sclerosis, cortical thickening, sequestrum/involucrum formation. KHE is indolent without systemic inflammation.
**Differential for Pattern 2 (Cortical Excavation)**		
Avulsive Cortical Irregularity	Cortical defect in the metaphysis.	Classic location (posteromedial distal femur); history of athletic activity; self-limiting; sclerotic base. KHE can occur in various locations.
Tufted Angioma / Spindle Cell Hemangioma	Can cause cortical depression/erosion.	Typically presents with an obvious, palpable soft tissue mass adjacent to the bone defect. KHE Pattern 2 is strictly intraosseous/subperiosteal without a discrete soft tissue mass.
Periosteal Desmoplastic Fibroma	Cortical depression.	Marked sclerotic border/rim around the lesion. KHE excavations often have irregular, serrated bases without a thick sclerotic rim.
Other Vascular Mimics		
Intraosseous Hemangioma	Vascular tumor.	More common in adults (spine/skull). In long bones, shows coarse “polka-dot” or “jail-bar” vertical striations, not moth-eaten.
Intraosseous Lymphangioma	Vascular anomaly in bone.	Well-defined cystic/sieve-like lesion, often with sclerotic margins; no enhancement on contrast CT/MRI. KHE shows enhancement.
Gorham-Stout Disease (GSD)	Progressive osteolysis.	Causes massive, contiguous bone resorption (“vanishing bone”) respecting no boundaries; not focal excavations.
Pseudomyogenic Hemangioendothelioma	Multifocal lytic lesions.	“Lantern-cluster” morphology. Typically metaphyseal/cortical; rarely involves the epiphysis (unlike KHE).

#### Pattern 1: permeative/moth-eaten infiltration (*n* = 7)

**Fig. 2:**
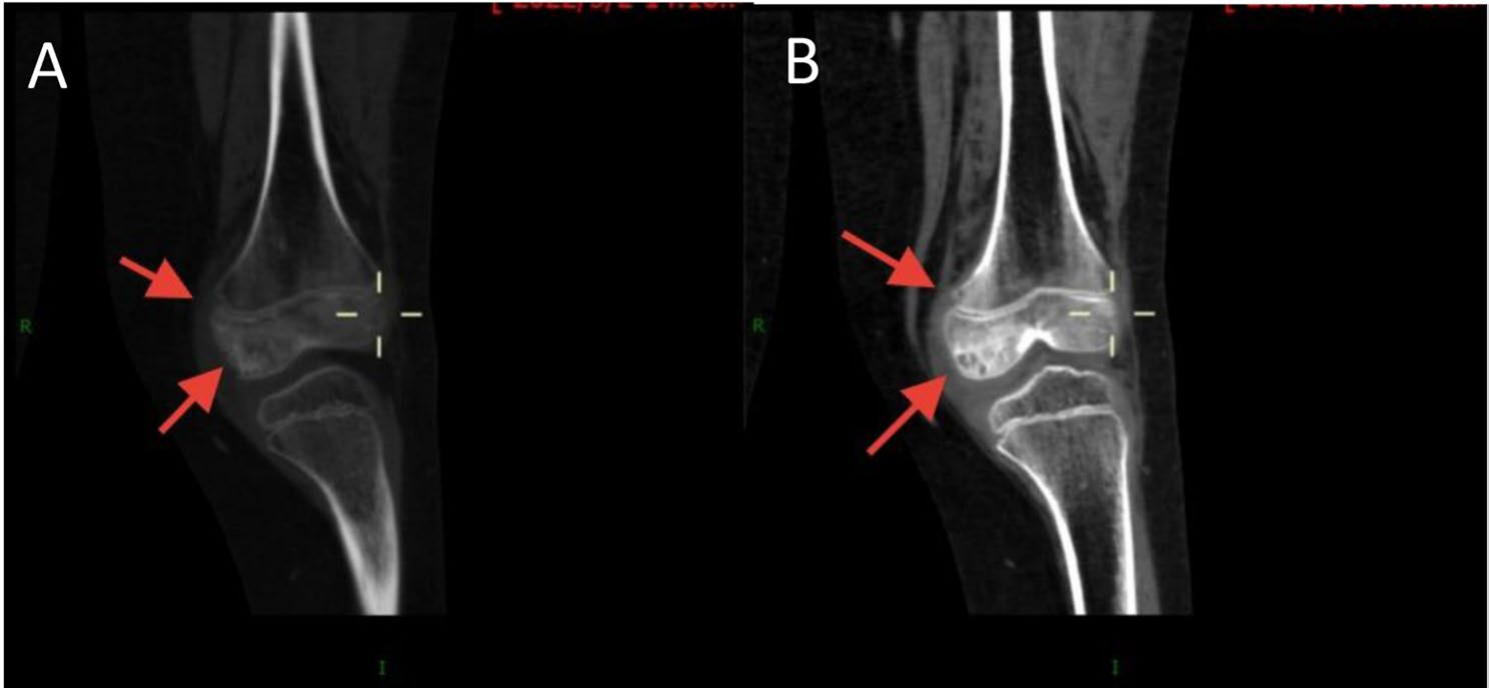
Permeative/moth-eaten infiltration pattern (Pattern 1) of KHE in an 8-year-old male with an abnormal gait. (**A**) Coronal CT image (bone window) of the right proximal femur demonstrates multiple small, dotted, and moth-eaten osteolytic lesions (white arrows) with ill-defined margins, involving both the epiphysis and metaphysis. (**B**) Sagittal CT reconstruction shows a prominent tubular or channel-like lucency (black arrow) traversing the metaphysis, a characteristic finding in this pattern. Note the absence of periosteal reaction or a soft-tissue mass. (**C**) Coronal T2-weighted MRI shows heterogeneous hyperintensity throughout the affected epiphysis and metaphysis

The most common pattern, observed in seven patients, was characterized by infiltrative osteolysis with permeative and moth-eaten features (Fig. 2). These lesions presented as clusters of small, dotted, or punctuated lucencies with ill-defined margins, lacking a distinct solid or cystic component. A key feature of this pattern was the consistent involvement of both the epiphysis and the adjacent metaphysis, often crossing the physis. In all seven cases (100%) within this group, multiplanar CT reconstructions revealed linear or serpentine channel-like lucencies, described as “tubular formations,” coursing through the trabecular bone. These tubular structures occasionally traversed the cortex to create small perforations but did not produce a significant extraosseous mass. Sclerotic rims were generally absent, although mild reactive sclerosis was noted around some tubular channels. Critically, no aggressive periosteal reaction was observed in any case. On MRI, these lesions demonstrated isointense to mildly hypointense signal on T1-weighted images and heterogeneously hyperintense signal on T2-weighted and PD-FS images.

#### Pattern 2: cortical excavation (*n* = 5)

**Fig. 3:**
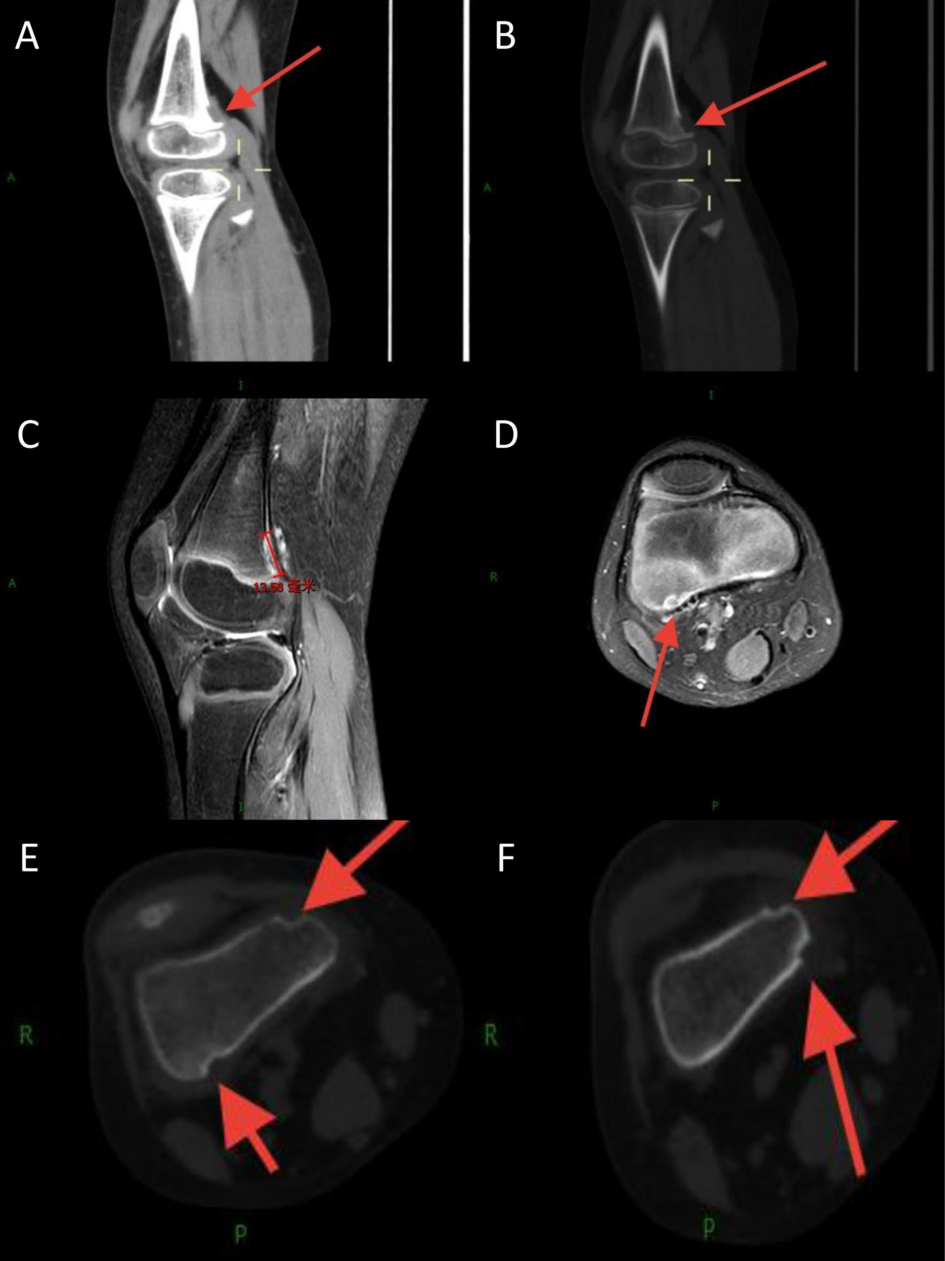
Cortical excavation pattern (Pattern 2) of KHE in a 7-year-old male with chronic knee pain. (**A**) Axial CT image (bone window) of the distal femur shows a focal, saucer-like excavation of the posteromedial cortex (white arrow), with an irregular base. (**B**) Sagittal CT reconstruction better delineates the “apple bite” appearance of the cortical defect (black arrow). (**C**, **D**) Sagittal and axial proton density-weighted fat-suppressed (PD-FS) MRI sequences show high signal intensity at the site of the cortical erosion (white arrows), with minimal adjacent marrow edema and no associated soft-tissue mass

The second pattern, identified in five patients, manifested as focal, saucer-like cortical defects or excavations, creating an appearance reminiscent of an “apple bite” on tangential views (Fig. 3). These lesions were predominantly located in the metaphysis of long bones (4/5 cases), with only one case involving the epiphysis. The base of the excavation was typically irregular or serrated, and the underlying medullary bone showed minimal signal or density changes. Unlike the permeative lesions of Pattern 1, these cortical defects were more localized. On cross-sectional imaging, they appeared as scalloped erosions of the outer cortical surface. There was no associated periosteal reaction, sclerosis, or evidence of fracture. In the two cases with contrast-enhanced MRI, mild enhancement was noted at the margin of the defect. Minimal adjacent soft-tissue edema or fat stranding was present in two cases, but no discrete enhancing soft-tissue mass extended from the bone.

### Histopathological findings

**Fig. 4:**
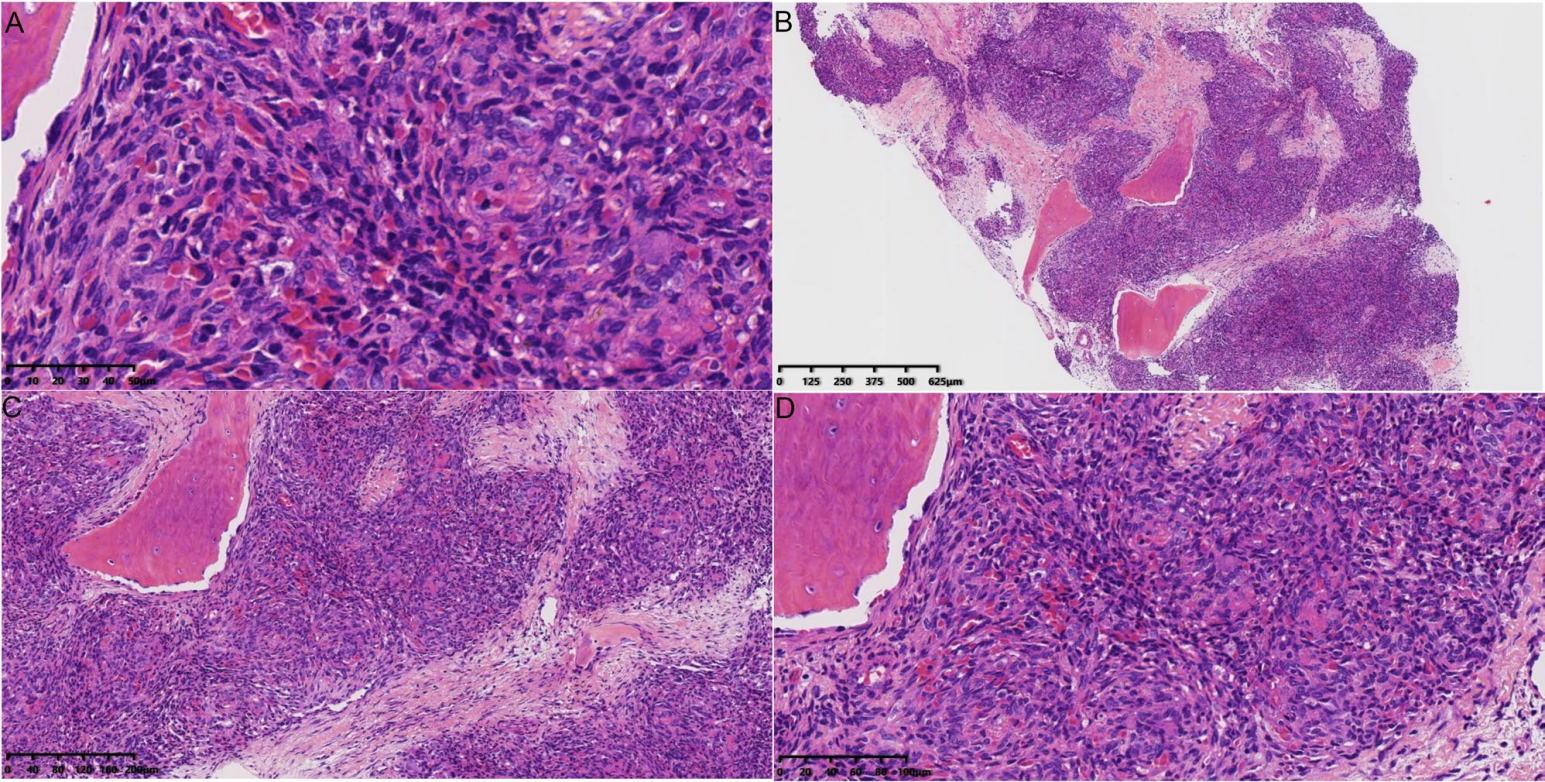
Histopathological features of primary osseous KHE. (**A**) Low-power view (H&E, ×40) shows infiltrative nodules of tumor cells destroying and replacing the native trabecular bone, separated by fibrous tissue. (**B**) Medium-power view (H&E, ×100) demonstrates the characteristic lobular architecture, with spindled tumor cells forming ill-defined nodules containing slit-like vascular spaces filled with red blood cells. (**C**) High-power view (H&E, ×200) reveals spindled endothelial cells arranged around crescent-shaped vascular lumina. (**D**) Higher magnification (H&E, ×400) shows bland, short spindled cells with eosinophilic cytoplasm, some containing intracytoplasmic vacuoles. Nuclear atypia and mitotic figures are absent

**Fig. 5:**
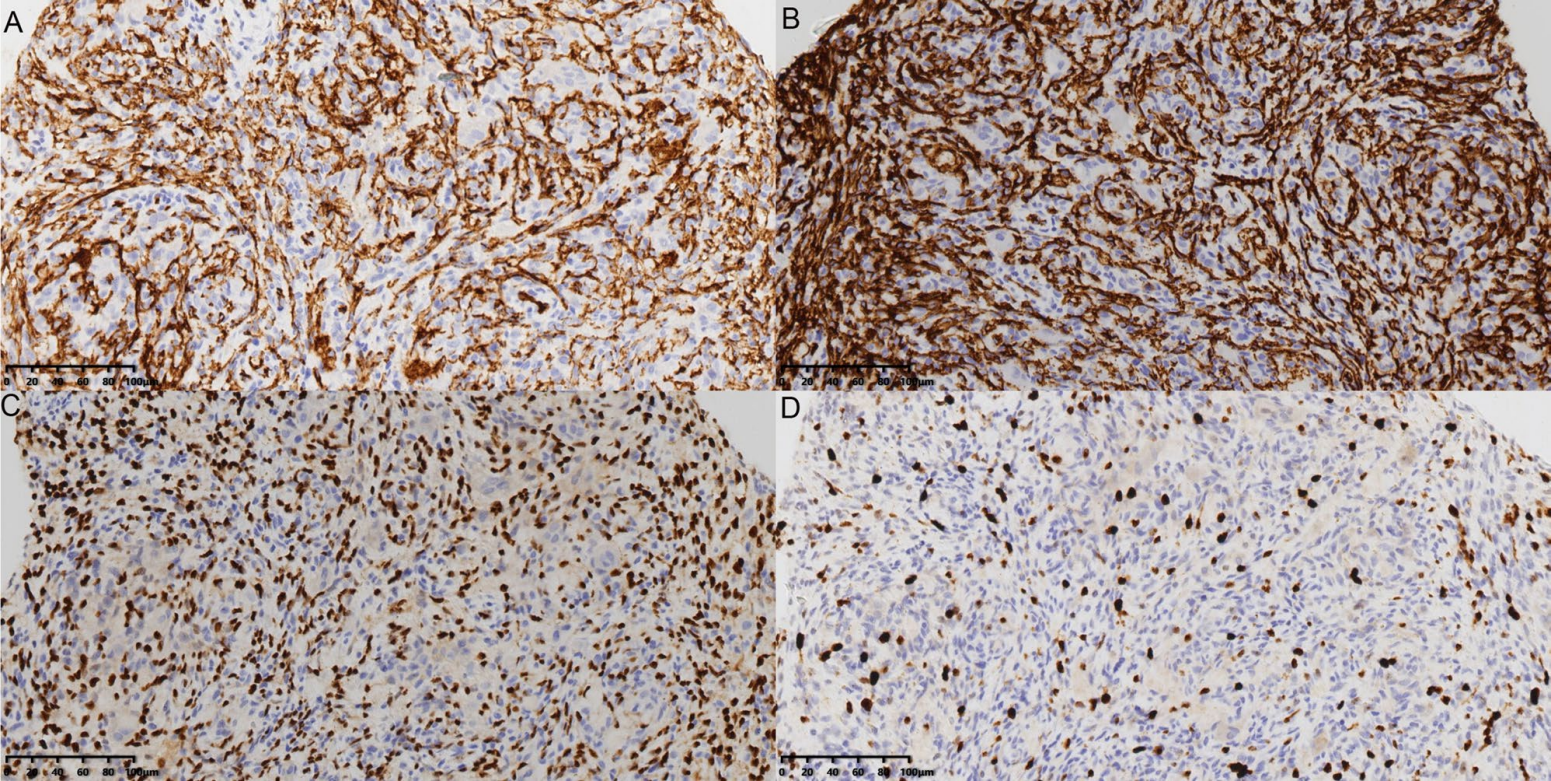
Immunohistochemical findings supporting the diagnosis of KHE. (A) CD31 staining (×200) shows strong positive expression on the cell membrane and cytoplasm of the endothelial cells. (B) CD34 staining (×200) demonstrates similar positive membranous/cytoplasmic expression. (C) ERG staining (×200) shows nuclear positivity, confirming vascular endothelial lineage. (D) Ki67 staining (×200) reveals nuclear positivity with a cell proliferation index of approximately 15%, consistent with a vascular tumor of intermediate malignancy

**Fig. 6:**
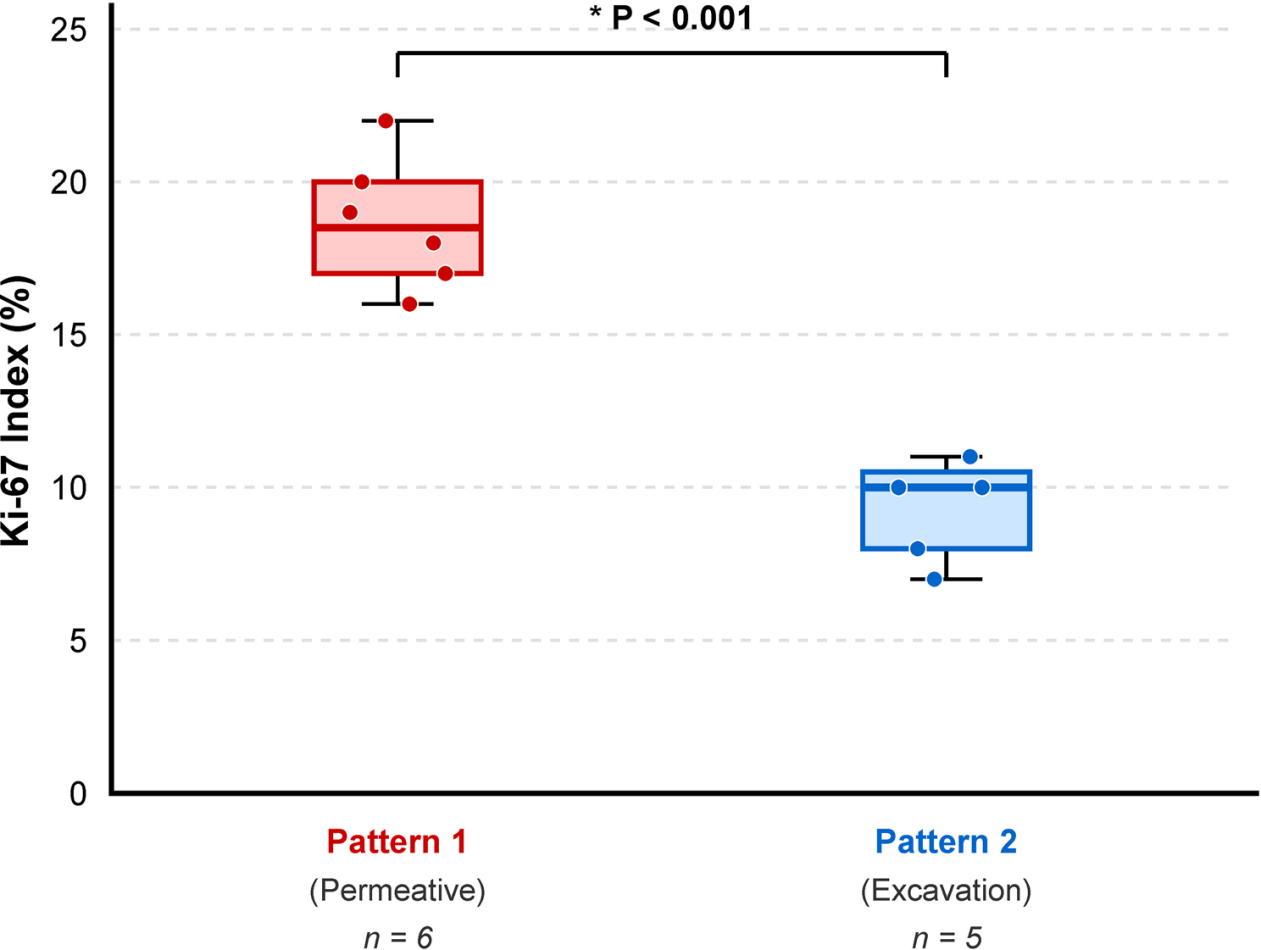
Comparison of Ki-67 proliferation index between the two radiological patterns. Box and whisker plot demonstrating significantly higher Ki-67 expression in the permeative/moth-eaten pattern (Pattern 1) compared to the cortical excavation pattern (Pattern 2) (*P < 0.001). The central line represents the median, the box edges represent the 25th and 75th percentiles, and the whiskers extend to the minimum and maximum values. Note: The single mixed-pattern case was excluded from this statistical comparison to ensure group homogeneity

Histopathological examination of biopsy specimens from all 12 patients confirmed the diagnosis of KHE. Microscopically, the tumor was characterized by an infiltrative growth pattern, destroying and replacing the normal trabecular bone. It was composed of poorly defined nodules of neoplastic cells separated by fibrous connective tissue (Fig. 4A). The nodules consisted of tightly packed, bland-appearing spindled endothelial cells that formed irregular, slit-like, or crescent-shaped vascular channels containing red blood cells (Fig. 4B and C). The tumor cells were short, spindled, or polygonal, with eosinophilic cytoplasm and indistinct cell borders. Some cells contained intracytoplasmic vacuoles, occasionally with engulfed erythrocytes. Nuclear atypia was minimal, and mitotic activity was very low or absent (Fig. 4D). IHC analysis demonstrated strong and diffuse positivity for CD31 and D2-40 (podoplanin) in the neoplastic cells, confirming their dual vascular and lymphatic endothelial differentiation. The Ki-67 proliferation index was significantly different between the two radiological patterns (see Fig. 5). Pattern 1 (permeative) tumors (*n* = 6; excluding the single mixed-pattern case) showed a mean Ki-67 index of 18.7% ± 2.2%, whereas Pattern 2 (excavation) tumors (*n* = 5) had a mean Ki-67 index of 9.2% ± 1.8% (*P* < 0.001) (Fig. 6).

## Discussion

This study characterizes the clinical and radiological features of a rare cohort of 12 pediatric patients with primary KHE strictly confined to the osseous compartment. Our analysis identified two distinct imaging patterns—permeative/moth-eaten infiltration and cortical excavation—which appear to represent the earliest manifestations of this disease. These findings contribute significantly to the understanding of osseous KHE, particularly in differentiating it from other pediatric bone lesions.

A striking finding in our cohort is the marked male predominance (11:1) and the complete absence of KMP or cutaneous lesions. This contrasts with larger, more heterogeneous KHE cohorts, which typically report a slight male predominance and a high incidence of KMP (up to 70%) and skin involvement (up to 75%) [8, 12]. The absence of these systemic features in our patients is likely attributable to the small volume and isolated intraosseous nature of the lesions. It is well-established that the risk of KMP correlates with lesion size (typically > 8 cm) and involvement of deep tissues like the retroperitoneum or mediastinum [8, 18]. The lesions in our study were small, reinforcing the hypothesis that purely osseous KHE represents a low-volume, early stage of the disease that has not yet reached the critical threshold to trigger systemic consumptive coagulopathy.

Pathologically, Kaposiform hemangioendothelioma (KHE) shares a continuous spectrum with tufted angioma (TA), a concept reflected in the WHO classification of soft tissue and bone tumors (5th edition) which suggests they may represent different manifestations or stages of the same tumor [19]. While both entities exhibit vascular nodules, TA typically presents as discrete ‘cannonball’ tufts of capillaries within the dermis or subcutaneous tissue [12, 20]. In contrast, KHE demonstrates a more aggressive, infiltrative lobular growth pattern composed of spindled endothelial cells forming slit-like vascular channels, often involving deeper tissues [12, 20]. In our osseous cases, the histology consistently demonstrated the infiltrative sheets characteristic of KHE rather than the discrete tufts of TA, supporting the diagnosis of KHE despite the unusual intraosseous location.

Our two identified patterns may represent different biological behaviors. The significant difference in Ki-67 proliferation indices between Pattern 1 (18.4%) and Pattern 2 (9.2%) suggests that the permeative pattern represents a more biologically active and proliferative form of the disease compared to the more indolent cortical excavation pattern. The permeative/moth-eaten pattern (Pattern 1), with its epiphyseal-metaphyseal involvement and tubular formations, suggests an infiltrative process spreading through medullary vascular channels. This pattern shares similarities with the six primary bone KHE cases reported by Kuo et al., which also described permeative lesions [14]. The cortical excavation pattern (Pattern 2) suggests a more indolent, pressure-eroding process originating from the periosteal or cortical surface. This morphology resembles a case described by Boccara et al. [10].

### Differential diagnosis

The imaging features of primary osseous KHE necessitate a broad differential diagnosis. While we must distinguish it from inflammatory conditions like osteomyelitis and Langerhans Cell Histiocytosis (LCH), a focused comparison with other vascular osseous tumors is crucial given the recent WHO classification.

### Differential diagnosis from other primary vascular bone tumors

#### Hemangioma of bone

Typically presents as a coarse, vertically striated ‘polka-dot’ or ‘jail-bar’ appearance in vertebral bodies or a radiating ‘sunburst’ pattern in flat bones [21]. Histologically, it consists of dilated, blood-filled spaces lined by flat endothelium, unlike the spindled cells of KHE.

#### Epithelioid hemangioma of bone

Often presents as well-defined lytic lesions with sclerotic margins [22]. Pathologically, it features epithelioid endothelial cells with abundant eosinophilic cytoplasm and vacuolization, distinct from the spindled morphology of KHE.

#### Epithelioid hemangioendothelioma of bone

A malignant tumor that appears as multifocal lytic lesions (‘lantern-cluster’) [23]. Histologically, it shows cords or nests of epithelioid cells in a myxohyaline stroma, often with the WWTR1-CAMTA1 fusion, which is absent in KHE.

#### Angiosarcoma of bone

A high-grade malignancy with aggressive osteolysis and cortical destruction [24]. Pathologically, it displays significant atypia, pleomorphism, and multilayering of endothelial cells, contrasting with the bland cytology of KHE.

For the **permeative/moth-eaten pattern (Pattern 1)**, the primary non-vascular differential includes pediatric malignancies and aggressive inflammatory conditions. **Ewing sarcoma** can present with a permeative pattern but is almost invariably associated with a large soft-tissue mass and an aggressive, lamellated, or sunburst periosteal reaction, features absent in our KHE cohort [25]. **Langerhans cell histiocytosis (LCH)** can cause lytic lesions, but classic LCH lesions are more well-defined (“punched-out”) with beveled edges and are often associated with a solid periosteal reaction [26]. Chronic **osteomyelitis** may show lytic changes, but typically demonstrates prominent periosteal reaction, cortical thickening, and potentially a sequestrum or abscess formation [27].

For the **cortical excavation pattern (Pattern 2)**, the differential includes benign and tumor-like conditions. An **avulsive cortical irregularity** is a common “do-not-touch” lesion, typically occurring at the posterior medial aspect of the distal femoral metaphysis; however, these are typically self-limiting and have a classic location [28]. **Gorham-Stout disease (GSD)**, or “vanishing bone disease,” is characterized by progressive osteolysis mediated by lymphatic proliferation but leads to massive, regional bone resorption rather than the focal lesions seen in our cohort [29].

### Clinical implications

Our findings have significant clinical implications. Radiologists encountering a pediatric lytic bone lesion exhibiting either a permeative pattern with epiphyseal-metaphyseal spread and tubular channels or a focal cortical excavation—especially in the absence of a soft-tissue mass or aggressive periosteal reaction—should include primary osseous KHE high in their differential diagnosis. Promptly suggesting this diagnosis can guide clinicians toward confirmatory biopsy and away from potentially inappropriate treatments for presumed malignancy or infection.

### Limitations

This study has several limitations inherent to its design. First, the retrospective nature and small sample size, although substantial for such a rare entity, may limit the generalizability of our findings. Second, being a tertiary referral center for orthopedic diseases, a selection bias may be present. Third, this study focused on the initial diagnostic imaging; a systematic analysis of follow-up imaging to document the natural history or treatment response of these two patterns was beyond the scope of this work and represents an important area for future research.

## Conclusion

In conclusion, primary Kaposiform hemangioendothelioma confined to bone is a rare entity in children that presents with two distinct radiological patterns: a permeative/moth-eaten infiltrative pattern and a cortical excavation pattern. These patterns also correlate with different proliferative potentials as indicated by Ki-67 levels. These presentations are typically not associated with the classic systemic signs of KMP or cutaneous lesions, making imaging the cornerstone of initial detection and diagnosis. Familiarity with these specific imaging features is essential for radiologists to distinguish KHE from its mimics and to facilitate early, appropriate management.

## Data Availability

The datasets used and/or analysed during the current study are available from the corresponding author on reasonable request.
